# HPV viral load in self-collected vaginal fluid samples as predictor for presence of cervical intraepithelial neoplasia

**DOI:** 10.1186/s12985-019-1253-2

**Published:** 2019-11-27

**Authors:** Malin Berggrund, Inger Gustavsson, Riina Aarnio, Julia Hedlund-Lindberg, Karin Sanner, Ingrid Wikström, Stefan Enroth, Matts Olovsson, Ulf Gyllensten

**Affiliations:** 1 Science for Life Laboratory (SciLifeLab), Department of Immunology, Genetics, and Pathology, Biomedical Center, Uppsala University, Box 815, 75108 Uppsala, Sweden; 2 Department of Women’s and Children’s Health, Uppsala University, 75185 Uppsala, Sweden

**Keywords:** Cervical cancer, Self-sampling, hrHPV, HPV viral load, CIN2+

## Abstract

**Objective:**

This study was performed to evaluate the use of high-risk HPV (hrHPV) viral load in screening tests for cervical cancer to predict persistent infection and presence of cervical intraepithelial neoplasia grade 2 or worse (CIN2+).

**Methods:**

We followed women between 30 and 60 years of age who performed self-sampling of vaginal fluid and subsequently a hrHPV test. Women who were hrHPV positive in their screening test repeated the hrHPV test 3–6 months later and were included in the present study.

**Results:**

Our results show that women with a persistent HPV16 infection had higher HPV viral load in their primary screening test than women with transient infections (*p* = 5.33e-03). This was also true for sum of viral load for all hrHPV types in the primary screening test (*p* = 3.88e-07). 48% of women with persistent HPV16 infection and CIN2+ had an increase in HPV16 titer in the follow-up test, as compared to only 20% of women with persistent infection but without CIN2+ lesions. For the sum of all hrHPV types, 41% of women with persistent infection and CIN2+ had an increase in titer as compared to 26% of women without CIN2 + .

**Conclusions:**

The results show that hrHPV viral load in the primary screening HPV test is associated with the presence of CIN2+ and could be used in triaging hrHPV positive women for different follow-up strategies or recall times. Serial testing of hrHPV viral load has the potential to distinguish women with CIN2+ lesions from women with persistent infection but without CIN2+ lesions.

## Introduction

Human papilloma virus (HPV) genital infection is associated with the development of cervical cancer. There are over 200 identified HPV types and 12 HPV types (16, 18, 31, 33, 35, 39, 45, 51, 52, 56, 58, and 59) are considered to be high-risk types (hrHPV) since they have substantial oncogenic properties [1]. Prevention of cervical cancer is achieved using prophylactic vaccination and by identifying precancerous lesions, classified as cervical intraepithelial neoplasia (CIN), through screening.

Cytology has been the most commonly used screening method to identify cervical lesions but due to its low sensitivity it is currently being replaced with hrHPV testing. HrHPV testing has a higher sensitivity than cytology but does not provide as high specificity. The specificity of hrHPV testing can be increased by triage with cytology or by repeating the HPV test in 4–6 months [2, 3]. About 40% of women that are hrHPV positive in their screening test have cleared their infection after 4–6 months [3]. The strategy of repeating the hrHPV test reduces the number of women that require follow-up and increases the specificity for identification of cervical intraepithelial neoplasia grade 2 or worse (CIN2+) [2]. Nevertheless, it would be optimal to use a single primary screening test to determine whether an hrHPV infection is likely to become persistent and result in cervical lesions or is likely to be a transient infection. At present, triage using cytology is recommended as follow-up for hrHPV positive women [4]. The low sensitivity of cytology means that some hrHPV positive women may have CIN2+ despite normal cytology in the triage test. To increase the specificity of triage, other biological markers have been suggested, including methylation of host or HPV genes, immunohistochemical staining of cervical smears for tumor markers, such as p16INK4a [5], and the use of HPV viral load.

An association between hrHPV viral load in cervical samples and severity of prevalent cervical disease was first described in 1999 [6] and replicated in numerous studies [7–9].

Most HPV infections are transient and a study of women 16–29 years of age has shown that 42% of HPV16 infections and 56% of HPV18 infections clear within one year [10]. Both for HPV16 and 18 baseline viral load was higher in persistent infections, and the authors concluded that hrHPV viral load is a marker for persistent infection. Using serial hrHPV viral load measurements it is possible to distinguish between regressing CIN2 and CIN3 lesions [11] and to predict progression to cervical cancer [12]. Previous studies based on 2–3 consecutive measurements have been able to predict the outcome of an hrHPV infection and the grade of CIN, indicating the potential of using HPV viral load in triage of HPV positive women [13].

The ability to predict risk of future cervical disease using hrHPV viral load was first shown in a retrospective nested case-control study [14–16]. These studies showed that HPV16 viral load can predict risk of developing CIN3 up to eight years before diagnosis. Finally, a nested case-control study also shown that hrHPV16 viral load can be a predictor of both persistence of infection and progression to CIN [17].

HrHPV viral load has also been studied for triaging of hrHPV positive women for cytology, colposcopy and clinical management, and high hrHPV viral load has shown a specificity of 96.4% and a sensitivity of 88% for distinguishing between women with high- and low-grade abnormal cervical cytology [18]. Using hrHPV viral load as compared to only hrHPV positivity, the number of women referred to treatment can be reduced by 52–81% [19]. HrHPV viral load threshold levels have been proposed for triage to immediate colposcopy and for reflex cytology [20]. A prospective study has suggested that in settings where cytology screening is not available, all HPV16/18 positive women should be referred to colposcopy and women with non-16/18 hrHPV infections should be triaged based on viral load [21]. However, other studies have found that HPV copy-number is associated with an increased risk of cervical abnormality, but that a single viral load estimate does not predict the risk of CIN, and concluded that HPV viral load is not a clinically useful biomarker [22]. Thus, while a large number of studies have been presented, the results have been conflicting or non-conclusive as to value of using HPV viral load to predict risk of cervical dysplasia.

Here we have used a cohort of women derived from two previous randomized studies, to investigate the ability to use the individual and combined viral load of 12 hrHPV types in the primary screening sample to predict persistence of hrHPV infection and risk of CIN2 + .

## Materials and methods

### Study population and samples

The data was derived from women between 30 and 60 years of age participating in two randomized intervention studies that were conducted in Uppsala County, Sweden, between 2013 and 2015 [3, 23]. The first randomized study included women between 30 and 49 years of age and had as aim to compare the detection rate of CIN2+ lesions based on histology in women performing repeated self-sampling of vaginal fluid for hrHPV testing with women following the regular screening program based on Pap smear cytology [3]. Only women in the intervention arm that followed the protocol of that study were included in the present analysis. The second randomized study included women between 50 and 60 years of age and had as aim to compare the detection rate of CIN2+ based on histology in women performing repeated self-sampling of vaginal fluid for hrHPV test with women sampled by medical personnel on the cervix for hrHPV test [23]. In the present study we only included women from the previous two studies that had performed self-sampling for hrHPV test according to study protocol. Women that were hrHPV-positive in their first sample were informed of the test result within 2 weeks after their sample was returned to the laboratory. These women were also informed that they would be asked to repeat the sampling in 4–6 months, and that they could contact a gynecologist in case of questions or symptoms. Women that were hrHPV positive in two consecutive HPV tests were referred to colposcopy and eventual biopsies. Women that were hrHPV negative in their first or second HPV test were referred back to the regular screening program. The follow-up period was 18 months from date of invitation.

### Self-sampling and sample processing

The procedure for self-sampling of vaginal fluid was based on using a silicon brush and the indicating FTA elute micro card™ and regular mail both for distribution of the sampling kit and return of the sample, as previously described [2]. The women were instructed to perform self-sampling of vaginal fluid using the Rovers® Viba-brush (Rover Medical Devices B.V., Oss, The Netherlands) and apply the vaginal fluid sample to the indicating FTA elute micro card™ (GE Healthcare, United Kingdom, art. no WB129308). Self-samples of vaginal fluid were returned to the HPV laboratory at Uppsala University by regular mail. The samples were processed using a dedicated automated laboratory system (easyPunch STARlet, Hamilton Robotics, Bonaduz, Switzerland) which collects each card, takes a photograph of the sample deposition area, identifies the regions with the highest amount of cellular material using a machine learning software, and then takes 4 punches from that area with a 3-mm diameter knife. The punches are deposited in a single well in a 96-well microtiter plate. DNA extraction from the punches was performed as described earlier [24].

### HPV DNA typing

HPV testing was performed using the clinically validated, real-time PCR-based, hpVIR test [25, 26]. This test detects and quantifies HPV16, 31, 35, 39, 51, 56 and 59 as individual genotypes, HPV18 and 45 in one group and HPV33, 52 and 58 as a second group. The test also detects and quantifies a human single copy nuclear gene (HMBS), which serves as a control for that the samples contain sufficient amounts of cellular material for the test to be informative, and a reference to which the HPV copy number can be related, i.e. for normalization of the HPV copy number. The limit of detection (LOD) for hpVIR was 10 copies per PCR for both the nuclear single copy gene HMBS and HPV.

### Colposcopy and histology

Gynecological examinations and colposcopies with biopsies were performed at the Clinic of Obstetrics and Gynecology, and histology at the clinic of Pathology and Cytology, both at Uppsala University Hospital, Uppsala. The outcome was the number of women with CIN2+ based on histology according to the SNOMED classification code diagnosed during the 18 months follow-up period from date of invitation. We also included squamous cell carcinoma, adenocarcinoma and adenosquamous carcinoma as outcomes.

### Statistical methods

Statistical calculations were performed and figures generated using R version 3.4.3 [27]. Significance levels for comparison between transient infections, persistent infections without CIN2+ and persistent infections with CIN2+ were calculated using the two-sided rank-based Spearman test (Wilcoxon) and *p*-values were adjusted for multiple testing using Bonferroni correction, and *q*-values< 0.05 considered significant. Odds ratios (OR) with 95% confidence intervals were calculated with the “oddsratio” function from the “epitools”-package (version 0.5–10) [28], and *p*-values were calculated with Fishers exact test. Receiver operating characteristic (ROC) curves were generated for women with transient infections and women with persistent infections and CIN2+ lesions. A general linear model was built using 50% of the samples for a 5-fold cross-validation training using the “caret”-package (version 6.0–78) in R [29]. The remaining 50% of the samples were used as test set to which the model from the training set were applied. The training set and the test set were chosen to contain the same frequency of cases and controls. ROC (reporter operator characteristic) curves, AUC (area under ROC curve), sensitivity and specificity were then generated using the pROC-package (version 1.10.0) [30].

All tests were performed for both hrHPV copy number, representing the amount of virus per sample (and PCR) and hrHPV titer, i.e. hrHPV copy number normalized by HMBS copy number, representing the amount of HPV per number of cells in the sample.

## Ethical approval

The study was approved by the Regional Ethics Committee in Uppsala (Dnr. 2012/099).

## Results

### Study characteristics

This study included women from two previous randomized studies whom had a positive hrHPV screening test and a follow-up hrHPV test in 4–6 month based on a second self-sample [3, 23]. From these two studies a total of 667 women performed two consecutive hrHPV tests, had histology available, and were considered eligible for the present analysis. In these women, a total of 752 hrHPV infections were detected in the primary screening test, with HPV16 being the most prevalent type and also the HPV type most likely to be present in the two consecutive hrHPV tests (HPV16 persistence; 76%, non-HPV16 persistence; 20–61%) (Table 1). Among women with persistent infection, 191 had or developed a CIN2+ histology during the 18-month follow-up period. The analysis was performed for both hrHPV copy number and hrHPV titer, i.e. hrHPV copy number normalized for amount of human cellular material.

**Table 1:** Type-specific hrHPV infections for the 667 women with two consecutive HPV results in the study. In total, 752 hrHPV infections were identified

HPV type	I No of transient infections	II No of persistent infections without CIN2+ lesions	III No of persistent infections with CIN2+ lesions	Total no of infections
16	50	64	93	207
18/45	36	31	18	85
31	37	28	24	89
33/52/58	58	38	51	147
35	16	3	1	20
39	17	17	7	41
51	34	25	12	71
56	28	24	7	59
59	15	15	3	33
Total	291	245	216	752

### HrHPV titer as predictor of persistence of infection and CIN2+ histology

The women were divided into three groups depending on hrHPV status and histology; I) transient infections (i.e. those that cleared the infection in the 4–6 months between the first and second hrHPV test), II) persistent infections (those with two consecutive positive hrHPV tests) but no CIN2+ lesion within the 18 months follow-up period, and III) women with persistent infection and CIN2 + .

**Fig. 1:**
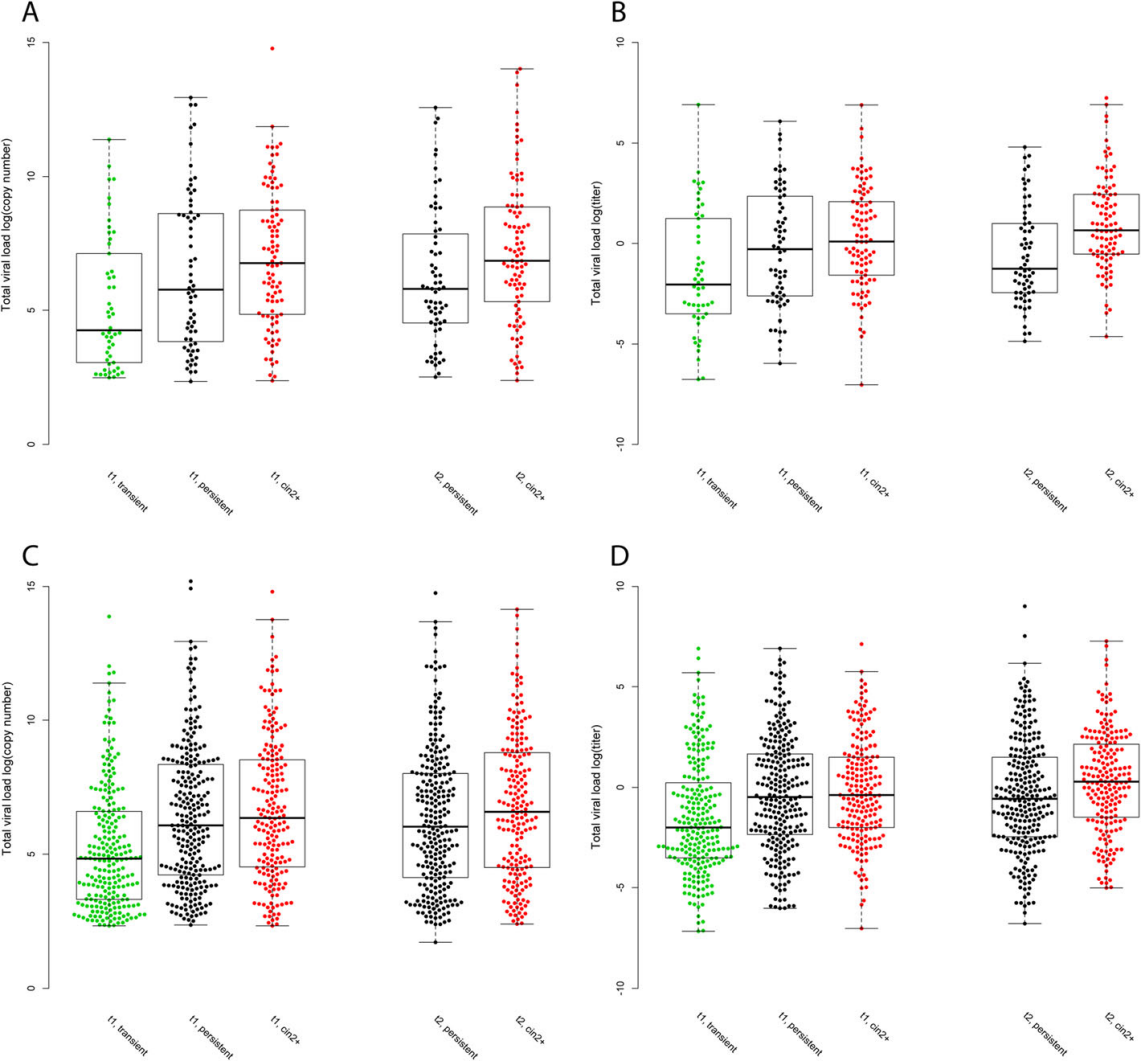
Combined scatter−/boxplot for log10 transformed HPV titer showing the distribution for transient infections (green), persistent infections without CIN2+ lesions (black) and persistent infection with CIN2+ lesions (red) in primary screening test (t1) and follow-up test (t2). The top and the bottom of the box represents the 25th and 75th percentile and the band inside the box the median value. The whiskers are calculated as 1.5x the interquartile range. A. HPV16 copy number. B. HPV16 titer. C. Total hrHPV viral load copy number. D. Total hrHPV viral load titer

HPV16 copy number and HPV16 titer in the primary screening test were both significantly higher in women with persistent infection, with and without CIN2+, as compared to women with transient infection (Bonferroni adjusted *p*-value = 1.42E-02 and 5.33E-03) (Fig. 1 A-B, Table 2). Similarly, both copy number and titer of the HPV33/52/58 group were significantly higher in women with persistent infection, with and without CIN2+, as compared to women with transient infection (Bonferroni adjusted *p*-value = 9.72E-07 and 1.82E-04) (Table 2). In the follow-up sample, HPV16 titer was significantly higher in women with persistent infection and CIN2+ as compared to women with persistent infection without CIN2+ (Bonferroni adjusted *p*-value = 2.29E-03) (Fig. 1B, Table 2).

**Table 2:** Median for type-specific hrHPV viral load and total hrHPV viral load and two-sided rank-based Spearman test with Bonferroni corrected *p*-values for the comparisons of hrHPV copy number and hrHPV titer in the primary screening and follow-up test between women with transient and persistent infection, with or without CIN2+ diagnosed during follow-up

HPV type	16	18/45	31	33/52/58	35	39	51	56	59	Total Viral Load
A. Baseline HPV self-sample test copy number										
Median										
Transient	71.6	84.7	109.2	53.2	1071.2	43.9	184.2	166.0	845.8	126
Persistent	593.1	112.1	364.4	751.7	327.7	376.8	442.1	153.2	336.5	497
CIN2+	866.2	92.6	438.4	688.8	13.4	140.4	9808.2	70.1	16,561.6	575
Bonferroni-corrected p-values										
Transient vs. Persistent	1.42E-02	1	5.89E-02	9.72E-07	1	0.3513	1	1	1	1.34E-08
Transient vs. CIN2+	5.33E-03	1	1.42E-01	1.82E-04	1	1	1	1	1	3.85E-07
Persistent vs. CIN2+	1	1	1	1	1	1	1	1	1	1
Follow up self-sample										
Median										
Persistent	329.7	433.3	243.0	732.2	982.9	1289.5	1392.7	212.0	125.0	415.9
CIN2+	945.7	84.4	131.7	534.7	429.9	626.2	4523.8	259.6	38,399.8	724.7
q-values, Bonferroni-corrected p-values										
Persistent vs. CIN2+	7.11E-01	1	1	1	1	1	1	1	1	1
B. Baseline HPV self-sample test titer										
Median										
Transient	0.13	0.09	0.12	0.05	1.47	0.05	0.17	0.41	1.58	0.13
Persistent	1.07	0.21	0.60	0.93	0.71	0.42	0.53	0.54	0.33	0.66
CIN2+	1.10	0.07	0.43	0.79	0.01	0.13	6.39	0.05	2.64	0.68
Bonferroni-corrected p-values										
Transient vs. Persistent	2.66E-02	1	0.23147	1.13E-04	1	1	1	1	1	5.82E-07
Transient vs. CIN2+	1.17E-02	1	1	4.44E-03	1	1	1	1	1	4.73E-06
Persistent vs. CIN2+	1	1	1	1	1	1	1	1	1	1
Follow-up self-sample										
Median										
Persistent	0.28	0.66	0.76	0.77	0.27	1.07	1.30	0.10	0.37	0.57
CIN2+	1.92	0.25	0.17	1.22	1.12	0.94	4.30	0.11	16.22	1.33
Bonferroni-corrected p-values										
Persistent vs. CIN2+	2.29E-03	1	1	1	1	1	1	1	1	0.14

Considering all hrHPV types, i.e. total hrHPV viral load in the sample, the copy number and titer in the baseline test was higher in women with persistent infection with or without CIN2+, in comparison to women with transient infection (Bonferroni adjusted p-value = 1.34E-08, 3.85E-07 and 5.82E-07, 4.73E-06) (Fig. 1 C-D, Table 2). There was no significant difference in total hrHPV copy number or titer in the follow-up sample between women with CIN2+ and those without CIN2+ (Fig. 1 C-D, Table 2).

### Distinguishing transient infection from persistent infection and persistent infection with CIN2+

Women with HPV16 infections were divided into four quartiles based on HPV copy number or titer in the primary screening test. First, we compared course of infection for women in the 1st titer quartile with women in the 2nd, 3rd and 4th titer quartiles, which gave an OR for persistent infection of 2.4, 3.9 and 3.9 for HPV16 infection and an OR of 2.2, 3.5 and 3.1 for all hrHPV titers (Table 3). For HPV copy number the OR was 1.9, 2.3 and 4.5 for HPV16 infection and 2.2, 3.5 and 3.1 for total hrHPV copy number, for the 2nd, 3rd and 4th titer quartiles, respectively. In comparison to the first quartile, the OR for distinguishing between a transient HPV16 infection and a persistent infection with CIN2+ was OR = 3.0, 3.4 and 3.1 for the 2nd, 3rd and 4th titer quartiles (Table 3). For total hrHPV titer, as compared to the first quartile, OR = 2.4, 2.9 and 2.3 for the 2nd, 3rd and 4th quartile (Table 3). For copy number, the OR were 2.1–3.6 for HPV16 and 1.4–2.4 for total hrHPV viral load (Table 3).

**Table 3:** Odds ratio with 95% confidence interval for HPV16 viral load and total hrHPV viral load for total hrHPV copy number and titer

	Quartile 1	Quartile 2	Quartile 3	Quartile 4
HPV16 copy number				
Range	10.5–54.9	56.0–404.2	414.0–4772.9	4952.2–2.6E+ 6
Median	23	131	1546	19,915
No of women with transient infection	20	13	11	6
No of women with persistent infection	32	39	41	45
Odds ratio, course of infection	NA	1.9(0.8–4.4)	2.3 (1.0–5.7)	4.5 (1.7–13.9)
OR, course of infection, *p*-value, fisher exact	NA	0.206	0.085	0.003
No of women with CIN2+	14	23	30	26
No of women without CIN2+	38	29	22	25
Odds ratio, CIN2+	NA	2.1 (0.9⎯5.0)	3.6 (1.6⎯8.5)	2.8 (1.2⎯6.5)
OR, CIN2+, *p*-value, fisher exact	NA	0.101	0.003	0.016
HPV16 titer				
Range	8.8E-04 ⎯ 7.1E-02	8.0E-02 ⎯ 0.65	0.65 ⎯ 7.26	7.31 ⎯ 994.68
Median	0.03	0.22	2.29	22.22
No of women with transient infection	22	12	8	8
No of women with persistent infection	40	40	44	43
Odds ratio, course of infection	NA	2.4 (1.0–5.8)	3.9 (1.6–10.7)	3.9 (1.6–10.4)
OR, course of infection, *p*-value, fisher exact	NA	0.059	0.004	0.004
No of women with CIN2+	13	26	28	26
No of women without CIN2+	39	26	24	25
Odds ratio, CIN2+	NA	3.0 (1.30⎯6.98)	3.4 (1.51⎯8.15)	3.1 (1.35⎯7.29)
OR, CIN2+, *p*-value, fisher exact	NA	0.015	0.005	0.008
Total hrHPV copy numbers				
Range	10.2–50.4	50.7–281.9	286.2–2594.1	2619.5–3.9E+ 6
Median	22.4	118.4	795.8	10,079.6
No of women with transient infection	82	68	45	33
No of women with persistent infection	86	99	122	133
Odds ratio, course of infection	NA	1.4 (0.9–2.1)	2.6 (1.7–4.1)	3.9 (2.4–6.4)
OR, course of infection, *p*-value, fisher exact	NA	0.015	4.55E-05	2.28E-08
No of women with CIN2+	33	43	53	62
No of women without CIN2+	134	124	114	104
Odds ratio, CIN2+	NA	1.4 (0.8⎯2.4)	1.9 (1.1⎯3.1)	2.4 (1.5⎯4.0)
OR, CIN2+, *p*-value, fisher exact	NA	0.240	0.017	4.22E-04
Total hrHPV titer				
Range	7.7E-04 ⎯ 6.0E-02	6.0E-02 ⎯ 0.4	0.4 ⎯ 3.9	3.9 ⎯ 1243.2
Median	0.02	0.15	1.12	17.5
No of women with transient infection	88	56	40	44
No of women with persistent infection	79	111	127	122
Odds ratio, course of infection	NA	2.2 (1.4–3.4)	3.5 (2.2–5.7)	3.1(1.9–4.9)
OR, course of infection, *p*-value, fisher exact	NA	5.92E-04	9.53E-08	1.26E-06
No of women with CIN2+	27	53	60	51
No of women without CIN2+	140	114	107	115
Odds ratio, CIN2+	NA	2.4 (1.4⎯4.1)	2.9 (1.7⎯4.9)	2.3 (1.4⎯3.9)
OR, CIN2+, *p*-value, fisher exact	NA	1.26E-03	5.73E-05	1.89E-03

### ROC curves for transient infections, persistent infections and CIN2+

**Fig. 2:**
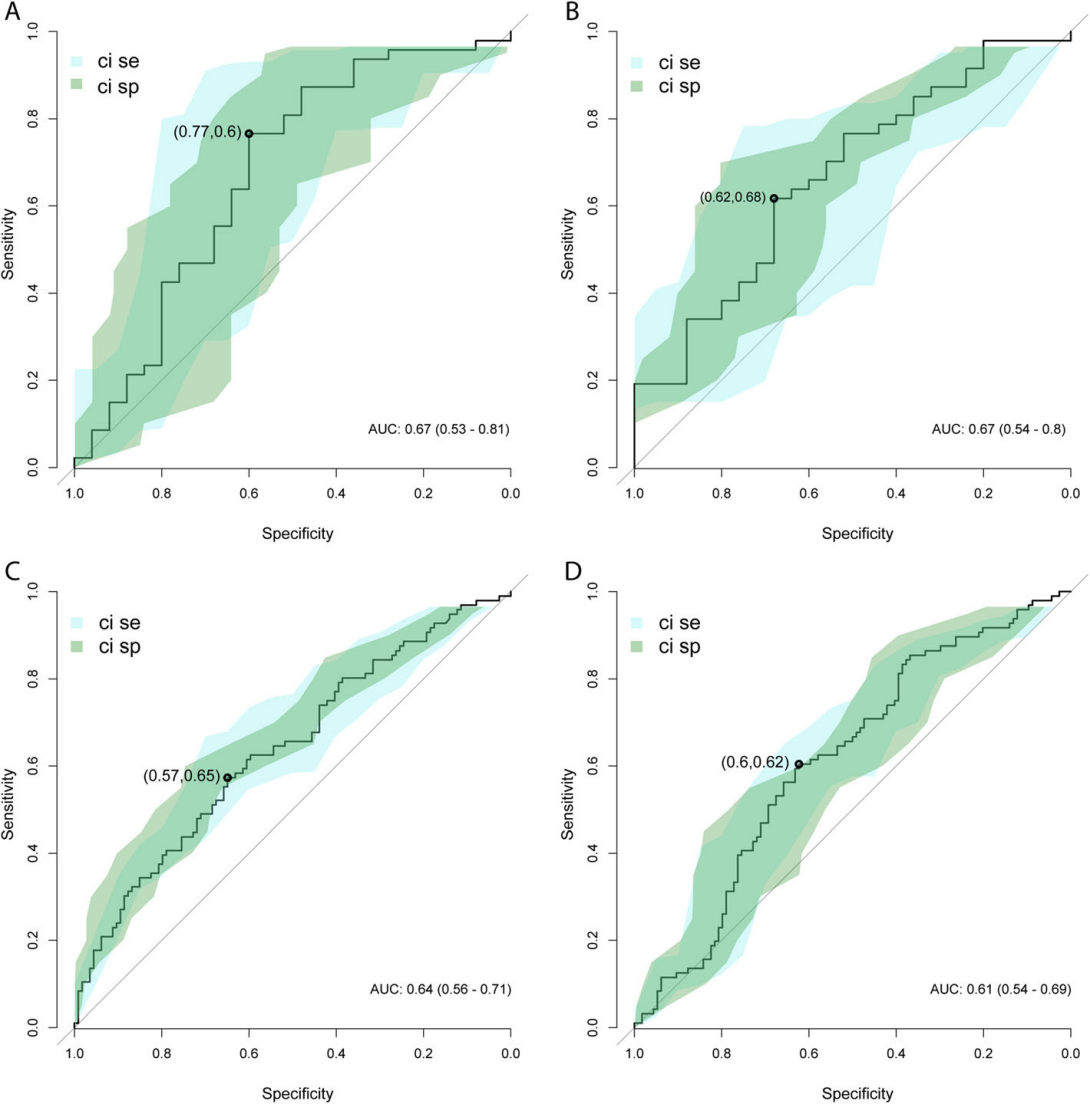
ROC-curves for women with hrHPV transient infections and women with hrHPV persistent infections with CIN2+ lesions. 50% of the samples were used for 5-fold cross-validation training in general linear modeling, and the models where then applied to the remaining 50% of the samples. 95% confidence intervals for sensitivity and specificity are shown as blue field (sensitivity) and green filed (specificity). Performance of trained model with validation set: A. HPV16 copy number, B. HPV16 titer, C. total hrHPV copy number, D. total hrHPV titer. The AUC with 95% confidence interval is given in each panel. Best point is indicated with black point and point estimates of performance. The best point is defined as the point on the ROC with closest to perfect classification, sensitivity and specificity 1.0

The samples with transient infections were used together with the samples with persistent infection and CIN2+ lesions to build a general linear model, using 50% of the samples for 5-fold cross-validation training. The remaining 50% of the samples were used as validation set to which the model from the training set was applied. The training and the validation sets were chosen to contain the same frequency of cases and controls. ROC curves for HPV16 copy number showed an AUC of 0.67 (0.53–0.81, 95% confidence interval) with best point sensitivity SE = 0.77 and specificity SP = 0.60 (Fig. 2 a). For HPV16 titer, AUC was 0.67 (0.54–0-80, 95% confidence interval) with SE = 0.62 and SP = 0.68 (Fig. 2 b). Total hrHPV viral copy number had an AUC of 0.64 (0.56–0.71, 95% confidence interval) with SE = 0.57 and SP = 0.65, and total hrHPV titer had an AUC of 0.61 (0.54–0.69, 95% confidence interval) and SE = 0.60 and SP = 0.62.

### Temporal HPV titer changes in women with persistent infection with or without CIN2+

As an indicator of viral load changes between serially collected samples, we compared the number of women that showed an increase of HPV titer between the baseline and follow-up hrHPV test, in women with CIN2+ and without CIN2+. An increase of titer was defined as an increase of 0.5 hrHPV copies per cell between the two sampling time points. For HPV16, 48% (45/93) of women with persistent infection and CIN2+ had an increase in HPV16 titer between the screening and follow-up test, as compared to 20% (13/64) of women with persistent infection but no CIN2+. The results were significantly different using two-sided Binomial test (*p*-value = 2.05e-09). For total hrHPV titer, 41% (79/191) of women with persistent infection and CIN2+ had an increase in hrHPV titer, as compared to 26% (64/248) of women with persistent infection but without CIN2+ (p-value = 5.11e-06).

## Discussion

We analyzed the association of hrHPV copy number and hrHPV titer in the primary screening test with persistence of infection and presence of CIN2+. Our results show that women with a persistent HPV16 infection, both with and without CIN2+ within the follow-up period, had significantly higher HPV viral load in the primary screening test than women with transient infections. This was also true for sum of viral load for the HPV33/52/58 group and the sum of all hrHPV types in the primary screening test. This is consistent with results from previous studies based on other study designs, and together provide evidence for that the copy number of several hrHPV types is increased during development of CIN.

Our results show that hrHPV titer can be used to predict both the course of an HPV infection (persistent/transient) and the presence of CIN2+. Based on HPV16 titer, women in the three highest copy number percentiles have OR = 2.4–3.9 for persistent infection and OR = 3.0–3.4 for CIN2+ presence within 18 months as compared to women in the lowest percentile. The results indicate that triaging based on hrHPV titer in the screening test could be used to stratify hrHPV positive women into those at high risk of CIN2+ presence and those likely to clear their infection. Women with high hrHPV titer could be directed for immediate clinical follow-up (colposcopy) without repeating the hrHPV test, while those with low titer could perform a second self-sampling and HPV test prior to clinical investigation.

Since the relationship between hrHPV viral load and CIN2+ risk differs between hrHPV types, an HPV test is required that is able to identify individual or specific groups of hrHPVs. Also, the test must enable quantification of hrHPV copy number and titer, such as the clinically validated hpVir test [26]. The most commonly used HPV tests in screening program (Hybrid Capture 2, Cobas HPV) only distinguish HPV16 and 18 from other hrHPV types, and do not report on viral copy number. Studies of three commercial assays (HC2, Cobas HPV, Aptima) have also shown that cross-reactivity with low-risk HPVs account for about 25% of false positive test results [31, 32]. Application of such test will most likely reduce the predictive power of using hrHPV titer.

Several previous studies have shown the potential of using serial measurements of HPV titer to predict the outcome of the infection, and suggested that only two measurements would be sufficient [11–13]. We found a significant association of both HPV16 viral load and total hrHPV viral load in two consecutive samples with presence of CIN2. This indicates that serial testing for hrHPV provides an opportunity to distinguish between women with CIN2+ and those without CIN2+, based on hrHPV viral load.

Further studies are needed to establish the optimal titer threshold for stratification of HPV positive women to alternative clinical strategies. Hybrid Capture 2 RLU values, which is a semi-quantitative estimate, shows an association with grade of CIN [20]. In resource-limited areas, where cytology screening is not available, it has been suggested that HPV16/18 positive women should be referred to colposcopy, while non-16/18 hrHPV positive women could be triaged using HPV titer [21]. HPV16, 31, 33, 52 and 58 viral load has been reported to be elevated in patients with ≥ high-grade squamous intraepithelial lesion (HSIL) [33]. Nevertheless, some studies have shown that although high HPV viral load is associated with risk of cervical abnormalities, a single estimate of HPV viral load does not reliably predict risk of CIN due to large individual variation in HPV titer [22]. Such variation is either because individual women have different titer development, the samples are collected at different time points in the progression towards CIN, or because of differences between the methods used to estimate HPV amounts and viral load. In our study we did not detect a difference in predictive value between samples from the same woman collected at two time points. This indicate that a single HPV viral load estimate could be used for risk prediction. However, the two samples from each woman were collected within a relatively short time interval, and examining longer time intervals may show other results.

Some limitations of our study should be recognized. The short time span between the baseline and the follow-up hrHPV tests, due to the medical practice at the time point of the study, imply that we measure short-term persistence of an infection, as a proxy for persistent infection. The short time between the baseline and the follow-up test leads to an overestimate of the number of persistent infections, which would reduce the statistical power to detect an association between titer and persistence of infection as compared to using a longer time interval.

## Conclusions

We found that triaging with hrHPV viral load could be used to identify women with higher risk of persistent hrHPV infection and presence of CIN2+. Also, for HPV16 infection, HPV tests that identify individual HPV types and can quantify viral load can be used for triage with HPV positivity and viral load. Triaging on the hrHPV viral load could be used to identify women that are likely to clear their infection without intervention, and therefore only need to recalled within a suitable time limit to check their HPV status.

## Data Availability

The datasets used and/or analysed during the current study are available from the corresponding author on reasonable request.

## Abbreviations

ASCUS: Atypical squamous cells of undetermined significance
AUC: Area under curve
CIN: Cervical intraepithelial neoplasia
CIN2 +: Cervical intraepithelial neoplasia grade 2 or worse.
HC2: Hybrid Capture 2
HPV: Human papilloma virus
HrHPV: High risk Human papilloma virus
HSIL: High-grade squamous intraepithelial lesion
LSIL: Low grade squamous intraepithelial lesion
ROC: Receiver operating characteristic
VIA: Visual inspection
